# Consecutive false‐negative rRT‐PCR test results for SARS‐CoV‐2 in patients after clinical recovery from COVID‐19

**DOI:** 10.1002/jmv.26192

**Published:** 2020-07-06

**Authors:** Guan Wang, Na Yu, Weimin Xiao, Chen Zhao, Zhenning Wang

**Affiliations:** ^1^ Department of Radiology The First Affiliated Hospital of China Medical University Shenyang China; ^2^ Department of Respiratory and Critical Medicine The First Affiliated Hospital of China Medical University Shenyang China; ^3^ Department of Anesthesiology, Union Hospital, Tongji Medical College Huazhong University of Science and Technology Wuhan China; ^4^ Department of Otolaryngology The First Affiliated Hospital of China Medical University Shenyang China; ^5^ Department of Surgical Oncology and General Surgery The First Affiliated Hospital of China Medical University Shenyang China

**Keywords:** COVID‐19, false‐negative, rRT‐PCR, SARS‐CoV‐2

## Abstract

This study reviewed the serial real‐time reverse‐transcription polymerase chain reaction (rRT‐PCR) results of 37 patients admitted to our hospital in Wuhan, China, who had three or more sequential negative results before discharge. Of these 37 patients, 14 (~38%) had a positive rRT‐PCR result after a negative result during convalescence, and 5 (~14%) had a positive rRT‐PCR result after two consecutive negative results during convalescence. These results suggest that it may be necessary to require that patients have three consecutive negative results before discharge, to ensure that they do not spread infection among members of their household, or in the community. We believe that our study makes a significant contribution to the literature because it is not currently the standard of care to require patients to have three consecutive negative results before discharge. Our results suggest that a relatively high proportion of patients may continue to shed severe acute respiratory syndrome coronavirus 2 after they have clinically recovered, and thus may transmit the infection to others.

## Highlights

- In this study, 14% COVID‐19 patients after clinically recovered were found to have consecutive false‐negative rRT‐PCR test result. That indicated patients who met with discharge criteria with 2 consecutive negative results may continue to shed SARS‐CoV‐2 and contribute to potential infection transmission.

**Abbreviations**
COVID‐19: corona virus disease 2019
FN: false‐negative
rRT‐PCR: real‐time reverse‐transcription polymerase chain reaction
SARS‐Cov‐2: severe acute respiratory syndrome coronavirus 2
TN: true‐negative

## 1 BACKGROUND

Since December 2019, novel coronavirus disease (COVID‐19), caused by severe acute respiratory syndrome coronavirus 2 (SARS‐CoV‐2) infection, has widely spread to various countries and formed a global pandemic.^1^ By May 2020, 5 829 474 are confirmed all around the world and 360 776 died of COVID‐19.^2^ Pneumonia is the main manifestation with fever and cough, but critical subjects present dyspnea, reduced blood oxygen saturation, acute respiratory distress syndrome, and shock.^3^ Upper respiratory tract sampling and real‐time reverse transcriptase‐polymerase chain reaction (rRT‐PCR) were still routinely test to detect the virus.^4^ Convalescent patients with two consecutive negative results are considered to be no longer shedding the virus and can be discharged.^5^ However, some recovered patients have been retested positive after two negative test results,^6^, ^7^ leading to concerns that two consecutive negative results might be unreliable. This study aimed to determine the reliability of two consecutive rRT‐PCR tests.

## 2 METHODS

The study protocol was approved by the Ethics of Committees of The First Affiliated Hospital of China Medical University and the Union Hospital, Tongji Medical College, Huazhong University of Science and Technology. The requirement for informed consent was waived because the study was based on a retrospective review of medical records.

We retrospectively reviewed the records of consecutive patients with laboratory‐confirmed COVID‐19 admitted to the Wuhan Union Hospital, China from 9 February to 28 March 2020.

The treatment of patients in this study was in line with the Guidelines for the Diagnosis and Treatment of COVID‐19 Pneumonia published by the National Health Commission of the People's Republic of China.^5^ Patients discharged from the hospital after receiving systematic antiviral therapy were included in this study. The inclusion criteria were: (a) resolution of symptoms; (b) significant improvement in the amount of inflammation on the chest computed tomography scan; (c) three consecutively negative rRT‐PCR test results on samples collected at least 1 day apart. Given that recurrent positive rRT‐PCR results after discharge had been reported when two consecutively negative results were adapted as discharge criteria,^7^ additional rRT‐PCR tests after two consecutive negative results were performed in our ward. After excluding two patients without swab samples available due to invasive ventilation, and one patient receiving convalescent plasma therapy, 37 discharged patients were included in this study. Longitudinal rRT‐PCR test results of throat swabs were collected from the onset of clinical remission until discharge. False‐negative (FN) result was considered as a negative result between two positive results. Specifically, the negative result in the sequence “positive, negative, positive” was defined as a “single FN” result, and the consecutive negative results in the sequence “positive, negative, negative, positive” were defined as “consecutive FN” results, while, three consecutive negative results were considered true‐negative (TN) results and were used as the criterion for discharge.

## 3 RESULTS

**Figure 1:**
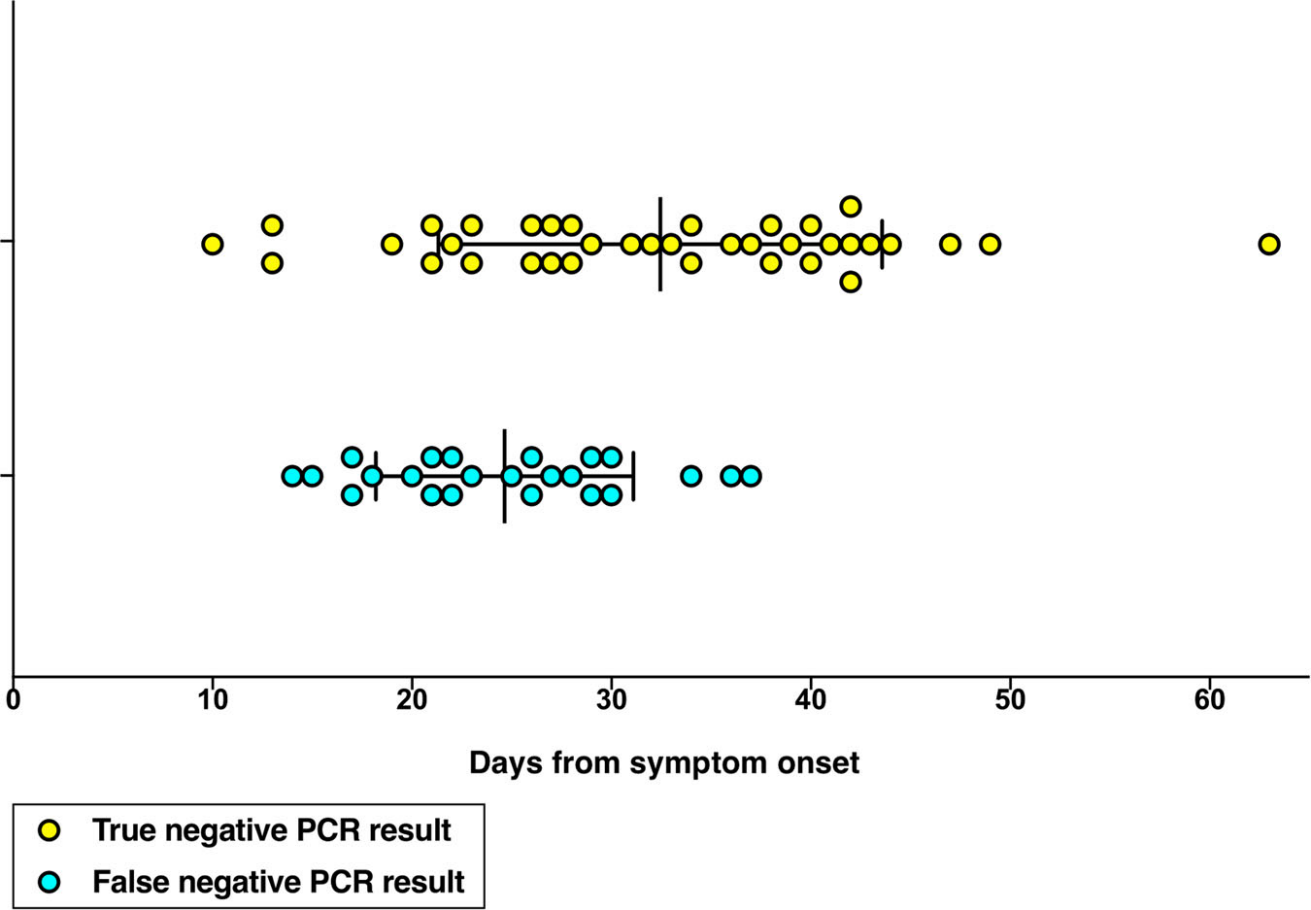
Time distributions of false‐ and true‐negative SARS‐CoV‐2 PCR results. PCR, polymerase chain reaction; SARS‐CoV‐2, severe acute respiratory syndrome coronavirus 2

**Figure 2:**
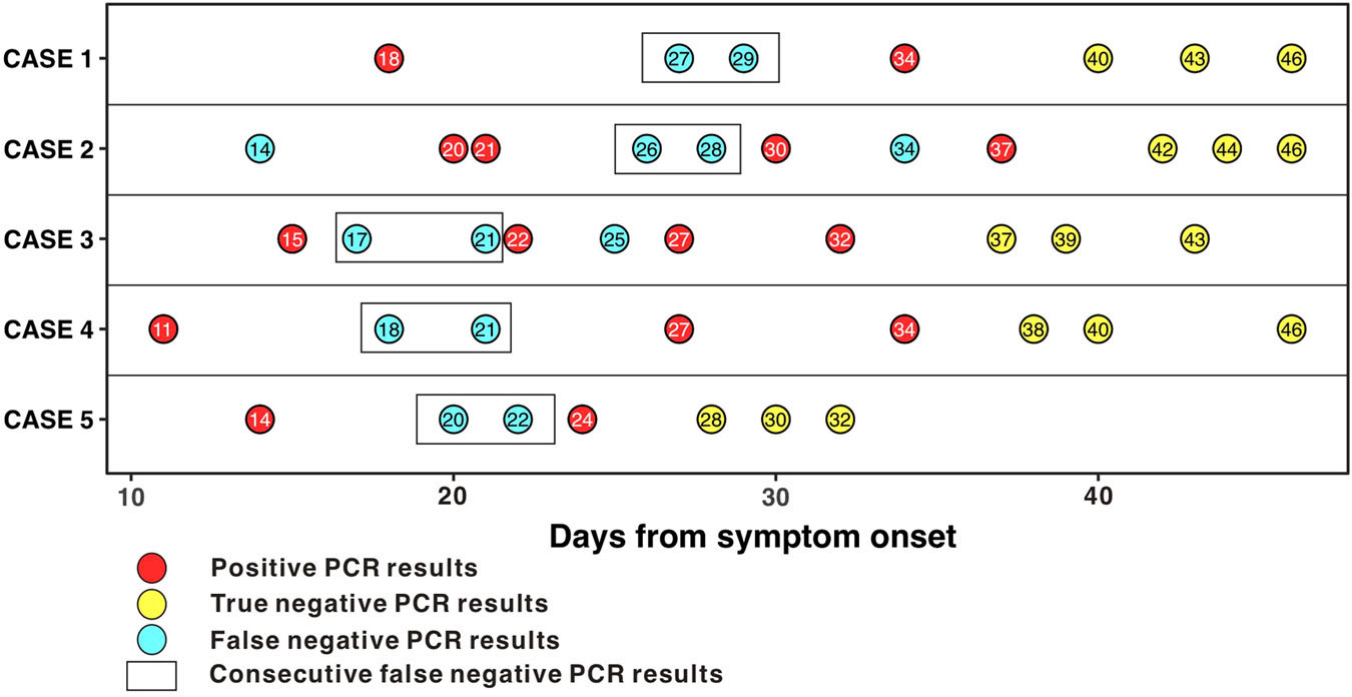
Timeline of SARS‐CoV‐2 rRT‐PCR results from clinical remission to discharge in five patients with two consecutive false‐negative results. rRT‐PCR, real‐time reverse‐transcription polymerase chain reaction; SARS‐C0V‐2, severe acute respiratory syndrome coronavirus 2

Thirty‐seven patients were included in this study, median age was 62 years old, 17 (45.9%) were male. The most common symptoms were fever (29, 78.4%), cough (21, 56.8%), shortness of breath (10, 27%), and fatigue (5, 13.5%). Hypertension, cardiac disease, and diabetes mellitus was found as comorbidities in seven, five, five patients, respectively, only two patients had pre‐existing pulmonary disease. 27 patients had three consecutive TN rRT‐PCR results, seven had four TN results for verification and three had five TN results for verification before discharge. Fourteen of the 37 patients (38%) had one or more FN result. The median time from symptom onset to a FN result was 25 days, ranging from 14 to 37 days; the median time from symptom onset to the first TN result was 33 days, ranging from 10 to 63 days (Figure 1). Nine (24%) patients had a single FN result, and five (14%) had two consecutive FN results. The sequences of rRT‐PCR results in the five patients with two consecutive FN results are shown in Figure 2.

## 4 DISCUSSION

Previous studies have revealed the occurrence of FN rRT‐PCR test results^6^, ^7^ thus at least two consecutive negative results are generally required for confirmation. However, this study revealed multiple patients with two negative results followed by a positive result, indicating that two consecutive negative rRT‐PCR results were insufficient as a discharge criterion. Several factors contributed to FN, including viral shedding depended on disease severity and decreased along with treatment, lower load in throat swabs than sputum samples,^8^ operator sampling skill, and accuracy. Moreover, SARS‐CoV‐2 shedding often persisted after clinical recovery.^9^, ^10^ Increasing the number of tests conducted before discharge is useful in identifying virus carriers. This study showed that two consecutive negative rRT‐PCR results did not rule out persistent infection. Thus, three consecutive negative rRT‐PCR results should be considered as a requirement for discharge.

Limitations of this study include the small number of cases, and the time variation of the sampling interval. Ongoing viral load and serum antibody monitoring are needed in future studies.

## CONFLICT OF INTERESTS

The authors declare that there are no conflict of interests.

## AUTHOR CONTRIBUTIONS

GW participated in the design of the study and draft the manuscript. NY performed the statistical analysis. WX participated in the acquisition, analysis, or interpretation of data. CZ conceived of the study and participated in its design and coordination and helped to draft the manuscript. ZW participated Critical revision of the manuscript for important intellectual content and supervised this study. All authors read and approved the final manuscript.

## ETHICS STATEMENT

The study protocol was approved by the Ethics of Committ ees of The First Affiliated Hospital of China Medical University and the Union Hospital, Tongji Medical College, Huazhong University of Science and Technology, the approval number is 2020191. The requirement for informed consent was waived because the study was based on a retrospective review of medical records.
